# The intellectual base and research fronts of IL‐18: A bibliometric review of the literature from WoSCC (2012–2022)

**DOI:** 10.1111/cpr.13684

**Published:** 2024-08-26

**Authors:** Zhongzhi Wang

**Affiliations:** ^1^ Department of Dermatology, Shanghai Fourth People's Hospital, School of Medicine Tongji University Shanghai China

## Abstract

Interleukin‐18 (IL‐18) is a vital pro‐inflammatory cytokine crucial for immune regulation. Despite its significance, bibliometric analysis in this field is lacking. This study aims to quantitatively and qualitatively assess IL‐18 research to construct its intellectual base and predict future hotspots. We conducted a thorough search on the Web of Science Core Collection for relevant publications between 1 January 2012 and 31 December 2022. English‐language articles and reviews were included. Visual analysis was performed using various tools including VOSviewer, Citespace, and Microsoft Excel. Our analysis covers interleukin‐18 (IL‐18) literature from 2012 to 2022, exploring research trends comprehensively. Key institutions like Yale University and Shanghai Jiao Tong University emerged as significant contributors. Prolific authors such as Kanneganti and Dinarello made notable contributions. Main focus areas included biology, medicine, and immunology. Co‐citation analysis highlighted influential works like Jianjin Shi. Hotspot keyword frequency cluster analysis revealed emerging themes like pyroptosis and psoriasis. Gene co‐occurrence clustering identified genes associated with immune regulation and inflammation. GO and KEGG pathway enrichment analysis provided insights into IL‐18‐related biological processes and pathways. Protein–protein interaction networks identified core proteins such as IL10 and TNF. Association disease analysis linked IL‐18 to various inflammatory, autoimmune, and metabolic disorders. This bibliometric review offers insights into IL‐18 research trends over the past decade, guiding future investigations and serving as a reference for researchers in this field.

## Graphical abstract

Clustered network map displaying the frequency of hotspot words related to IL‐18. VOSviewer software was used to conduct co‐occurrence cluster analysis on keywords, with a minimum occurrence frequency of five times per keyword. A total of 3279 keywords were cleaned, meaningless words were removed, synonyms were merged, and 171 keywords were selected and visualized on the map.

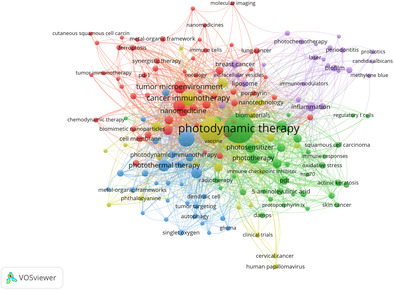

## 1 INTRODUCTION

Interleukin‐18 (IL‐18) is a cytokine with pro‐inflammatory properties that is vital in regulating immune responses. At its inception in 1989, it was identified as an interferon‐gamma (IFN‐γ) stimulant by T cells and natural killer (NK) cells.^1^ Since then, extensive research has been conducted to understand its mechanisms of action and functions. IL‐18 is predominantly produced by activated macrophages and dendritic cells; however, epithelial cells and keratinocytes are also capable of producing this hormone.^2^ It plays a crucial role in various biological processes (BP), including the regulation of cellular growth, innate and adaptive immunity, inflammation, and programmed cell death.^3^ IL‐18 exerts its biological effects by attaching to the IL‐18 receptor (IL‐18R), which is found in various cell types, such as T cells, B cells, NK cells, and macrophages. Upon binding to its receptor, IL‐18 triggers the activation of signalling pathways downstream, including the NF‐kappaB and MAPK pathways, leading to the synthesis of diverse cytokines and chemokines.^4^

The regulation of immune responses is critically influenced by the cytokines IL‐18 and IL‐18‐binding protein (IL‐18BP). Activated macrophages and dendritic cells produce IL‐18.^5^ Conversely, IL‐18BP is a natural inhibitor of IL‐18 that can regulate its biological function. The interaction between IL‐18 and IL‐18R is hindered by the binding of IL‐18BP to IL‐18, consequently blocking its biological effects. Multiple factors, including pro‐inflammatory cytokines, such as tumour necrosis factor‐alpha (TNF‐α) and interleukin‐1 beta (IL‐1β), regulate the production of IL‐18BP by inducing its expression.^6^ IL‐18BP can also be produced by various cell types, such as neutrophils, monocytes, and endothelial cells.

Maintaining the equilibrium between IL‐18 and IL‐18BP plays a crucial role in governing immune responses. An imbalance in this equilibrium is associated with different conditions, including inflammatory bowel disease (IBD), psoriasis, and systemic lupus erythematosus (LE).^7^ Therefore, it is crucial to comprehend the mechanisms underlying the regulation of IL‐18 and IL‐18BP and their disruption in illnesses to develop novel therapeutic strategies. Recent studies have primarily focused on the involvement of IL‐18 and IL‐18BP in the regulation of immune responses in cancer and microbial infections. IL‐18 and IL‐18BP can regulate the immune response to both cancer and microbial infections. Furthermore, an imbalance in the levels of these cytokines can facilitate tumour growth and the occurrence of microbial infections.^8^ Accordingly, it is crucial to comprehend the processes governing the regulations of IL‐18 and IL‐18BP in cancer and microbial infections.

Nevertheless, there is a lack of extensive investigations regarding publication patterns, influential authors and institutions, their collaborations, areas of expertise, and emerging patterns in IL‐18 research.^9^ Bibliometrics is the examination of academic publications using quantitative analysis. By analysing the author, keywords, research institutions, countries, published journals, and other information of the literature in the field, we can gain a deeper understanding of the research hotspots and development directions in that field. This information can then be used for strategic planning, policy formulation, and has been widely applied across various subject fields.^10^, ^11^, ^12^

Through bibliometrics, one can comprehensively analyse and evaluate the relevant research conducted during the present period, effectively organizing the context of IL‐18 research and gaining fresh perspectives in this domain.^13^ Currently, there is a lack of bibliometric analysis of IL‐18 research.

## 2 MATERIALS AND METHODS

### 2.1 Data collection

Journal publications have strong timeliness and can accurately reflect the progression and changes of a research topic. The Web of Science Core Collection (WoSCC)—a bibliographic database—is one of the biggest and most extensive electronic databases of scientific literature on a global scale.^14^ The documents obtained from this database ensure that the conclusions are reliable and credible. The WoSCC database was searched using the following search terms: ‘IL‐18’ OR ‘IL 18’ OR ‘IL18’ OR ‘IL‐1g’ OR ‘IL1F4’ OR ‘interleukin 18’ OR ‘IL‐18BP’ OR ‘IL18BP’ OR ‘IL 18BP’ OR ‘IL18BPa’ OR ‘interleukin 18 binding protein’, with the search restricted to the period from 1 January 2012 to 31 December 2022. The inclusion criteria encompassed articles and reviews that were relevant to the search while excluding letters, brief reports, book reviews, and similar materials. This search finally yielded 9753 articles. The data were used to analyse and visualize authors, institutions, countries, journals, co‐cited references, and keywords. Gene and disease data were sourced from the Citexs platform for big data analysis to visualize genes and diseases.

### 2.2 Data analysis and visualization

Bibliometric methods were employed to scrutinize the literature, utilizing powerful software tools tailored for such analyses. VOSviewer 1.6.18 and Citespace 6.1.6 software, crafted respectively by Professor Chaomei Chen of Drexel University^15^ and the Centre for Science and Technology Studies at Leiden University,^16^ stand as prominent examples of bibliometric analysis software leveraging the Java kernel. These tools facilitate the visualization of intricate co‐citation networks, unveiling collaborations and temporal trends among countries, institutions, and individuals. The nodes' size denotes the publication count, while the line thickness signifies link strength, and node colours denote distinct clusters or periods.

VOSviewer excels in visualizing complex co‐citation networks, while Citespace specializes in knowledge domain and emerging trend visual analyses, encompassing cluster analysis, journal double graph overlay, timeline graph, as well as reference and keyword citation burst detection. In Citespace, circle size corresponds to article or citation count, while circle colour indicates the publication year, with warmer tones representing more recent years. Thicker lines between circles signify higher co‐occurrence frequencies. These complementary tools offer distinct advantages and limitations.^17^

Furthermore, data visualization and analysis were conducted using Pajek, Scimago Graphica, and the R package Clusterprofiler^18^ STRING (www.string-db.org) served as a valuable resource for protein–protein interaction (PPI) information. The top 100 proteins, ranked by appearance frequency, underwent PPI network analysis via STRING, with resulting network data imported into Cytoscape software (https://cytoscape.org). Node degree values were computed to gauge network connectivity.

To delve deeper into key pathways and targets concerning IL‐18 regulation, GO classification, and KEGG pathway analysis were performed to categorize genes functionally and predict their biological roles. Figures were generated using R, employing KEGG pathway and GO enrichment analyses, with a corrected *p*‐value threshold of <0.05.

Data pertaining to genes and diseases were sourced from the Citexs platform for comprehensive big data analysis (https://www.citexs.com), facilitating the visualization and examination of present status, focal points, and research trends within this review.

## 3 RESULTS

### 3.1 Annual publication trend analysis

**FIGURE 1:**
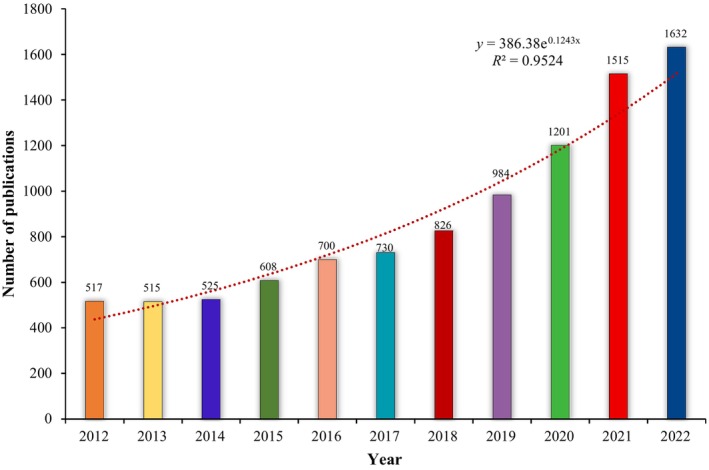
Trend in annual publication growth of interleukin‐18 (IL‐18) research.

From 2012 to 2022, the cumulative number of articles published on IL‐18 was 9753, whereas the average annual number of articles was 886.6, and the average growth rate was IL‐18. Figure 1 displays a consistent rise in the number of publications regarding IL‐18 from 2012 to 2022, suggesting a growing research inclination towards IL‐18 and signifying its importance in research. Meanwhile, we used the following formula to calculate the yearly increase in article publication: *y* = 386.38e0.1243*x* (*R* ^2^ = 0.9524), where *x* denotes year and y represents the total number of articles published annually. Figure 1 depicts the satisfactory curve fitting degree.

### 3.2 Regional relationship analysis

**FIGURE 2:**
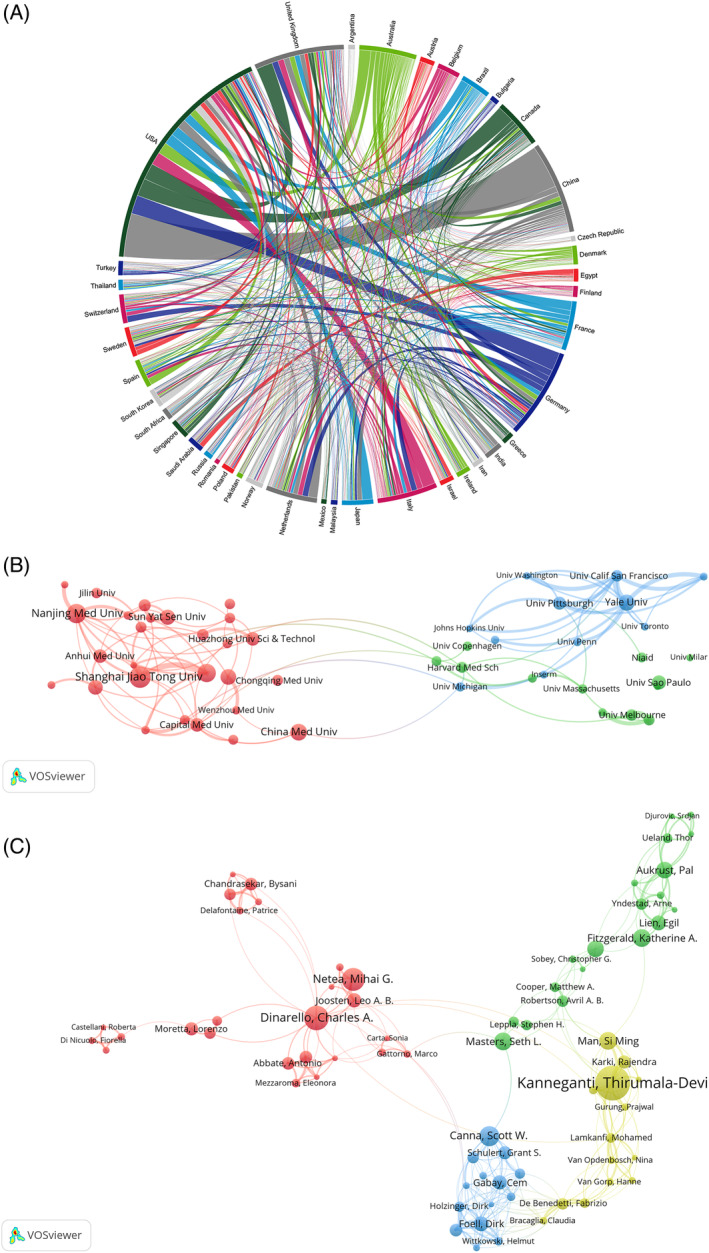
(A) Network map illustrating collaborative relationships between countries/regions. (B) Visualization of research institutions. (C) Visualization of researcher relations.

The chorddiag R package was used to visually analyse the publication regions among 119 countries, resulting in 9753 IL‐18‐related articles. Including authors who met the aforementioned criteria, a collaborative map of nations engaged in IL‐18 research was generated by establishing a threshold of 29 publications per country. Each segment of the outer curve represents each country, whereas the strength of collaboration among countries is indicated by the thickness of the connecting lines. Figure 2A depicts that the United States exhibits the highest inclination to engage in cooperation with other nations, with the collaboration between the United States and China being particularly intimate (Table 1).

**TABLE 1:** The top 10 countries/regions contributing to publications of IL‐18 research.

Rank	Country	Publications	Number of citations	Average number of citations	% of (9753)
1	China	2288	45,250	19.7770979	23.46%
2	United States	826	54,930	66.50121065	8.47%
3	Australia	216	12,182	56.39814815	2.21%
4	Brazil	98	2275	23.21428571	1%
5	Niaid	72	7076	98.27777778	0.74%
6	Sweden	69	1897	27.49275362	0.71%
7	Denmark	67	2373	35.41791045	0.69%
8	Norway	64	1756	27.4375	0.66%
9	French	56	2917	52.08928571	0.57%
10	Canada	55	2538	46.14545455	0.56%

Abbreviation: IL‐18, interleukin‐18.

### 3.3 Exploring institutional collaborations in IL‐18 research: A visual analysis

The VOSviewer software was used to perform a visualization analysis on the institutions that published research on IL‐18. A total of 8223 institutions have published 9753 IL‐18‐related articles. A collaboration network consisting of the leading 50 institutions was created by establishing a requirement of 50 published articles for an institution (Figure 2B). In the figure, clusters are represented by various colours, which were determined using the co‐citation network among institutions. This network combines highly cited relationships and generates a hierarchical diagram to illustrate the connections among institutions. The strength of collaboration among institutions is indicated by the thickness of the lines connecting the circles. Additionally, the circle size is directly proportional to the number of articles published by the institutions.

**FIGURE 3:**
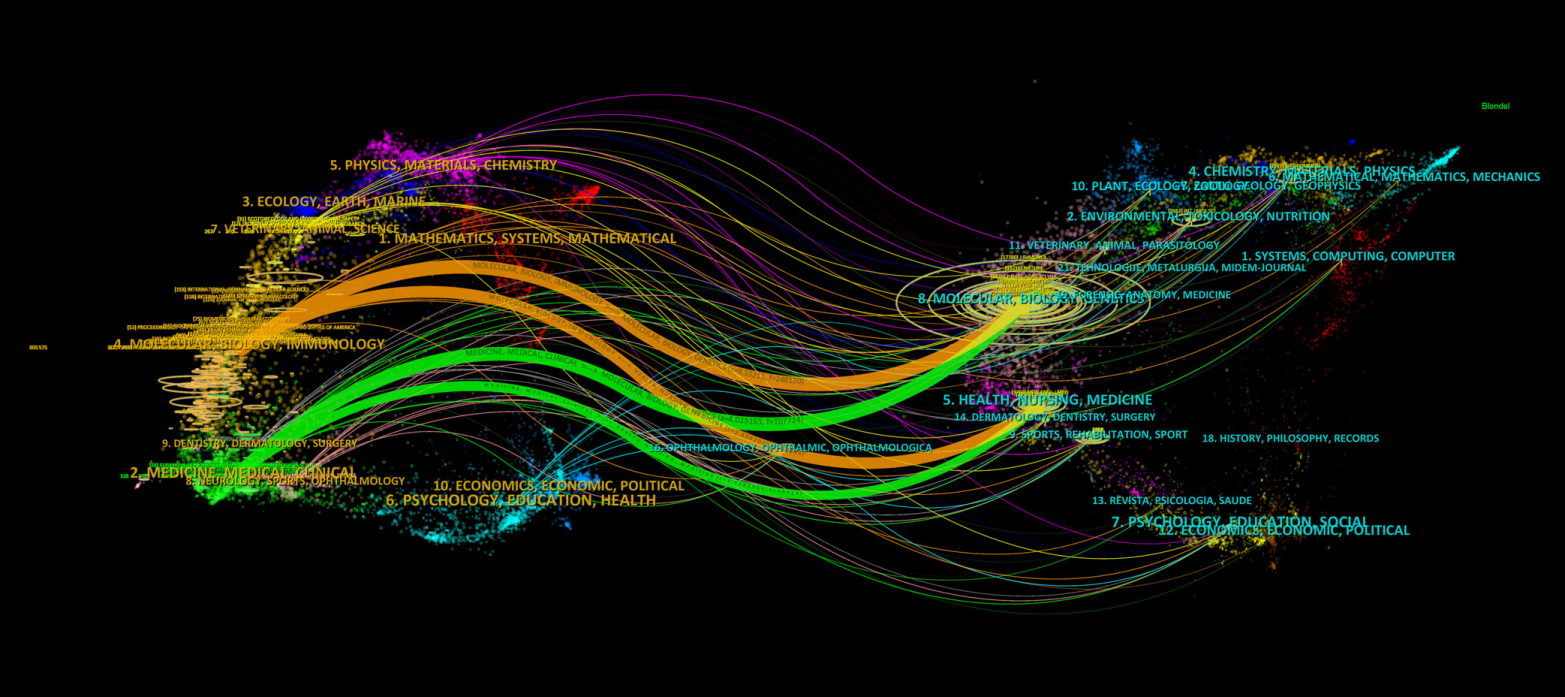
Dual‐map overlay of journals.

Based on Figure 3, Yale University exhibits the greatest inclination to cooperate with other establishments, whereas Shanghai Jiao Tong University leads in terms of publication count, securing the top position with 151 articles. The second and third highest number of publications was from Nanjing Medical University and Fudan University, with 135 and 127 articles published, respectively (Table 2).

**TABLE 2:** The top 10 institutions contributing to publications of IL‐18 research.

Rank	Institutions	Country	Aritide counts	Number of citations	Average number of citations
1	Shanghai Jiao Tong University	China	151	2730	18.0795
2	Nanjing Medical University	China	135	3351	24.8222
3	Fudan University	China	127	2975	23.4252
4	China Medical University	China	116	1300	11.2069
5	Yale University	United States	114	9780	85.7895
6	Zhejiang University	China	110	2572	23.3818
7	Sun Yat Sen University	China	107	2799	26.1589
8	Shandong University	China	101	1843	18.2475
9	University of São Paulo	Brazil	98	2275	23.2143
10	Huazhong University of Science and Technology	China	97	2132	21.9794

Abbreviation: IL‐18, interleukin‐18.

### 3.4 Study authorship relationship analysis

The VOSviewer software was used to perform a visualization analysis on 54,493 authors who published 9753 IL‐18‐related articles. To limit the number of nodes, we used a minimum publication threshold of 11 articles per author to generate the author collaboration graph for IL‐18 researchers. Figure 2C depicts the correlation between the number of articles published by the researchers and the size of the circles representing them, with circles coloured to indicate different clusters. Kanneganti, Thirumala‐Devi, Dinarello, Charles A., and Netea, Mihai G. are the leading authors in terms of publication count. The strength of collaboration among researchers is depicted by the thickness of the lines connecting the circles, with the closest collaboration observed between Kanneganti, Thirumala‐Devi, and Karki, Rajendra (Table 3).

**TABLE 3:** The top 10 most productive authors contributed to IL‐18 research.

Rank	Authors	Counts	Number of citations	Average number of citations
1	Kanneganti, Thirumala‐Devi	39	6294	161.3846
2	Dinarello, Charles A.	26	4092	157.3846
3	Netea, Mihai G.	24	1276	53.1667
4	Canna, Scott W.	21	1320	62.8571
5	Fitzgerald, Katherine A.	18	4248	236
6	Man, Si Ming	18	3334	185.2222
7	Masters, Seth L.	18	1155	64.1667
8	Aukrust, Pal	17	786	46.2353
9	Latz, Eicke	17	3558	209.2941
10	Foell, Dirk	15	547	36.4667

Abbreviation: IL‐18, interleukin‐18.

### 3.5 Mapping interdisciplinary connections in IL‐18 research: A journal overlay analysis

The journal overlay is divided into two sections: the left section represents the citing journals, whereas the right section represents journals that have been cited. The findings indicate the placement of IL‐18 research in relation to the primary fields of study. Figure 3 illustrates the citation connections between the left and right parts, with each journal represented by a point on the map. The connection paths offer insights into the interdisciplinary connections within the domain and effectively showcase the inception and progression of citations.

### 3.6 Research field analysis

**FIGURE 4:**
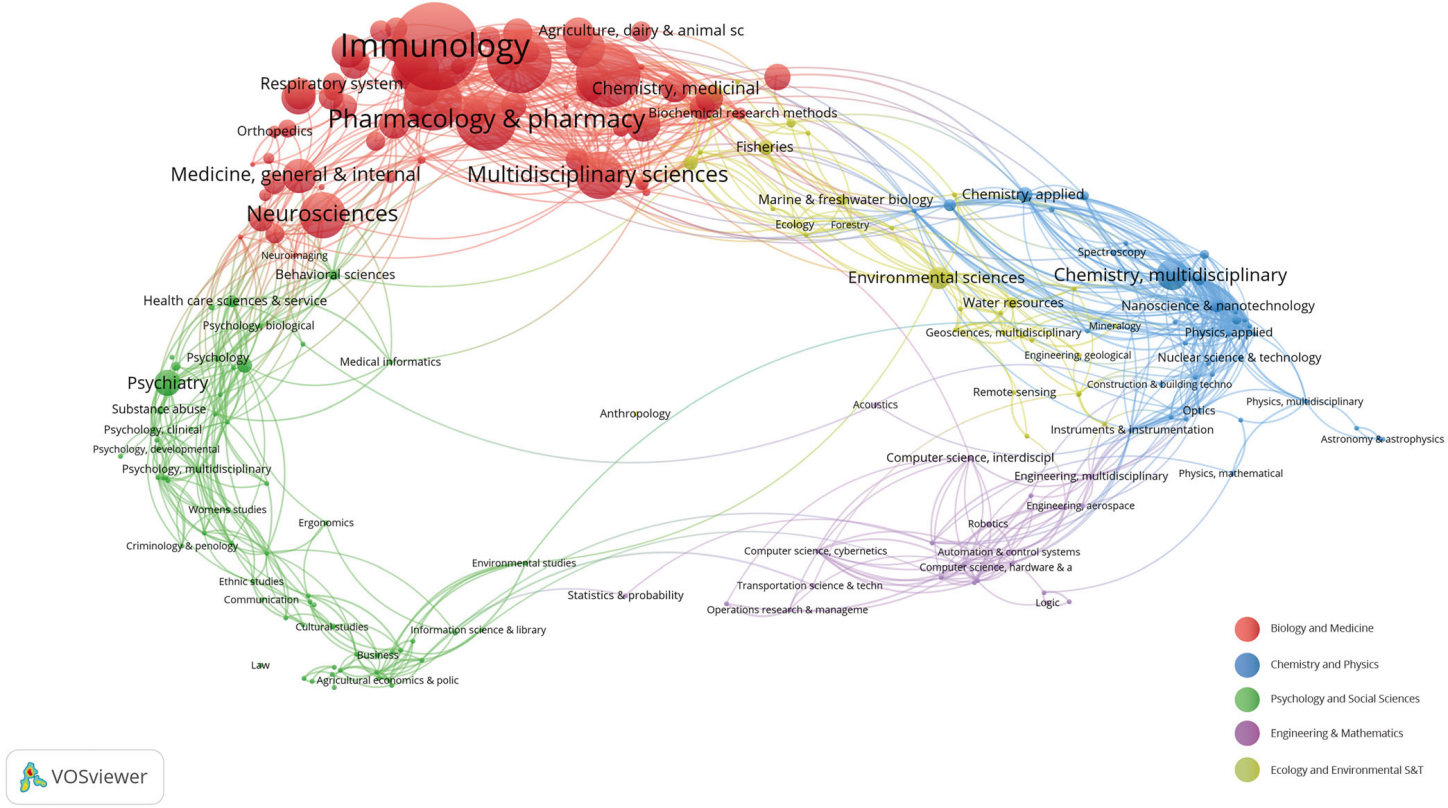
Clustered network map representing research field.

Statistical analysis was performed on the IL‐18 literature obtained from the WoSCC database, and the VOSviewer software was used for visual analysis. These analyses resulted in the clustering of 9753 IL‐18‐related articles into five major fields. Figure 4 demonstrates various field clusters represented by circles of varying colours. Most IL‐18‐related studies have focused on biology and medicine, particularly in the subfield of Immunology.

### 3.7 Co‐citation analysis of IL‐18 literature: Insights from CiteSpace

**FIGURE 5:**
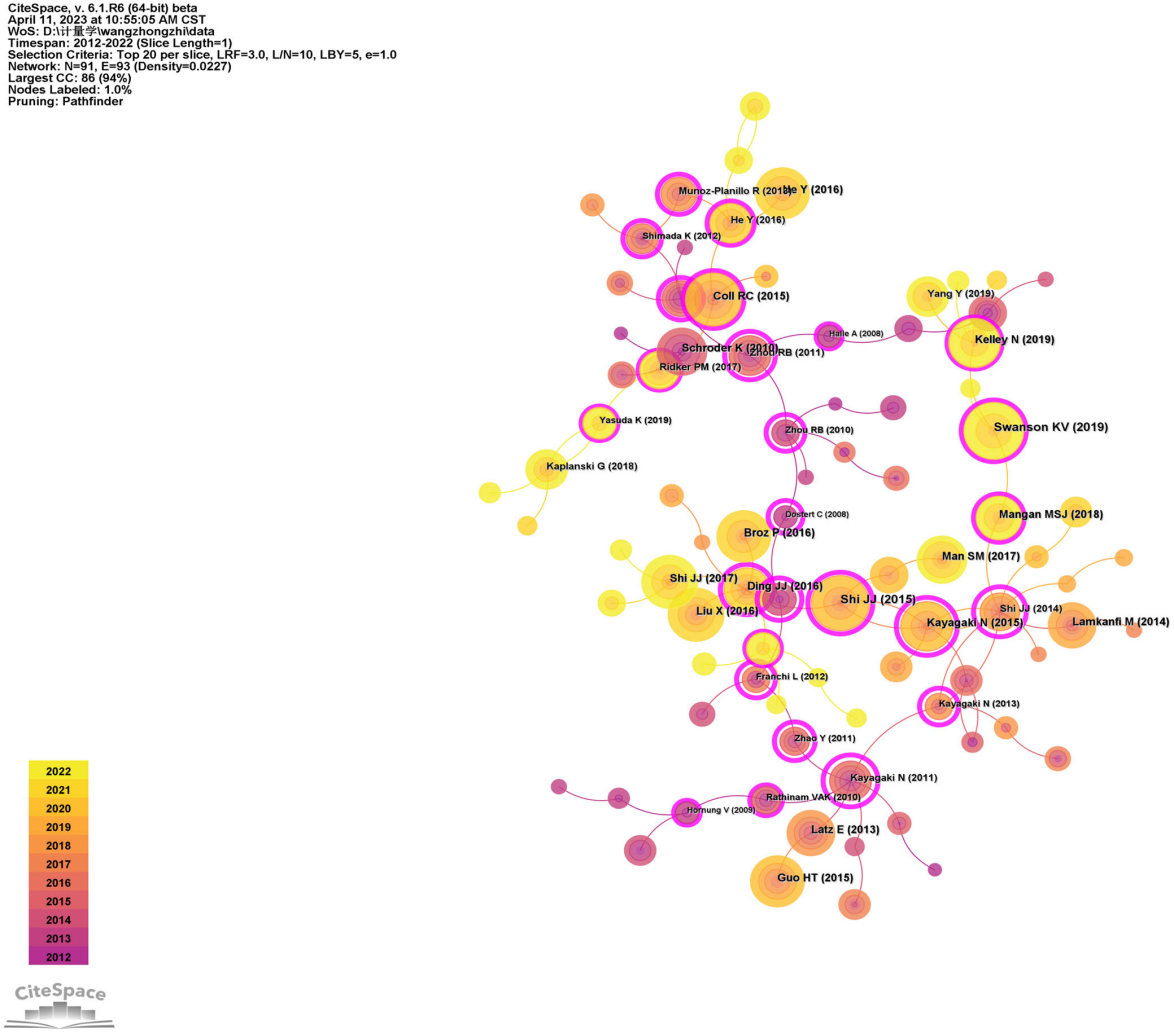
Clustered network map depicting co‐cited references in interleukin‐18 (IL‐18) research.

The co‐citation of IL‐18 literature from 2012 to 2022 was analysed using CiteSpace. The map includes 91 nodes and 93 links. The number of citations is directly proportional to the circle size. Purple indicates an earlier citation time, whereas yellow indicates a later citation time. The overlapping colours indicate that the article was cited in corresponding years. The connections among circles represent the co‐citation of literature. The nodes marked in magenta are key nodes with a centrality >0.1. Based on Figure 5, the article by Jianjin Shi has the highest number of citations (Table 4).

**TABLE 4:** The top 10 co‐cited references of IL‐18 research.

Rank	Title	Year	Author	Journal	Number of co‐citations	Centrality
1	Cleavage of GSDMD by inflammatory caspases determines pyroptotic cell death	2015	Jianjin Shi	Nature	259	0.54
2	The NLRP3 inflammasome: molecular activation and regulation to therapeutics	2019	Karen V. Swanson	Nat Rev Immunol	257	0.2
3	A small‐molecule inhibitor of the NLRP3 inflammasome for the treatment of inflammatory diseases	2015	Rebecca C. Coll	NAT MED	224	0.24
4	Inflammasome‐activated gasdermin D causes pyroptosis by forming membrane pores	2016	Xing Liu	Nature	223	0
5	Mechanism and regulation of NLRP3 Inflammasome Activation	2016	Yuan He	Trends Biochem Sci	220	0
6	Inflammasomes: mechanism of action, role in disease, and therapeutics	2015	Haitao Guo	Nat Med	214	0
7	Inflammasomes: mechanism of assembly, regulation and signalling	2016	Petr Broz	Nat Rev Immunol	214	0
8	Pyroptosis: gasdermin‐mediated programmed necrotic cell death	2017	Jianjin Shi	Trends Biochem Sci	213	0.08
9	Caspase‐11 cleaves gasdermin D for non‐canonical inflammasome signalling	2015	Nobuhiko Kayagaki	Nature	210	0.65
10	The NLRP3 inflammasome: an overview of mechanisms of activation and regulation	2019	Nathan Kelley	Int J Mol Sci	203	0.12

Abbreviation: IL‐18, interleukin‐18.

### 3.8 Hotspot keyword frequency cluster analysis

The VOSviewer software was used to perform co‐occurrence cluster analysis on keywords, with a minimum occurrence frequency of five times per keyword. A total of 3279 keywords were cleaned, meaningless words were removed, synonyms were merged, and 171 keywords were selected and visualized on a map.

**FIGURE 6:**
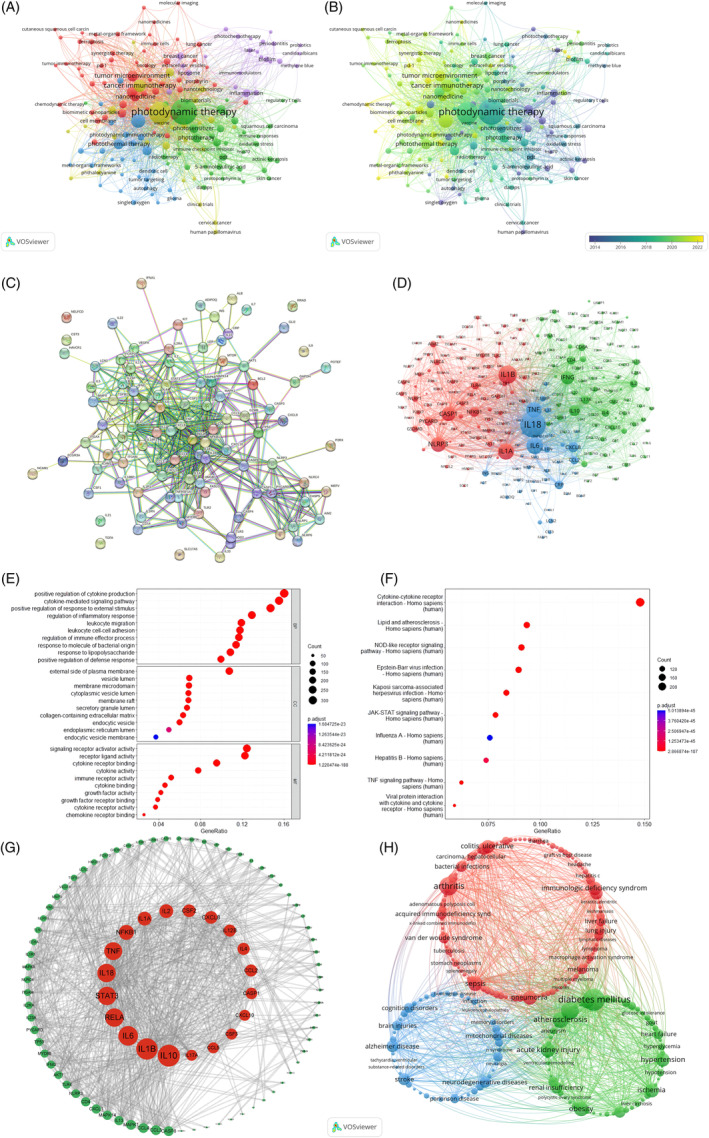
(A,B) Clustered network map showcasing hotspot word frequency. (C) Timeline view of co‐citation clusters. (D) Clustered network map of associated genes. (E) Clustered network map illustrating hotspot word frequency. (F) GO enrichment analysis plot. (G) KEGG pathway enrichment analysis plot. (H) Protein–protein interaction network construction analysis plot.

In Figure 6A, every node comprises a circular shape and a label. The circle size is directly proportional to the keyword's frequency, whereas the thickness of the connecting lines indicates the relationship strength among keywords. Clusters are formed by nodes of varying colours, with each colour representing a distinct research focus. Immunology is symbolized by the red cluster, psychology by the yellow cluster, chemistry by the green cluster, environmental sciences by the blue cluster, and interdisciplinary applications by the purple cluster.

Figure 6B illustrates that every node comprises a circular shape and a label. The circle size is directly proportional to the keyword's frequency, whereas the colour of each circle represents the average year of occurrence, as indicated by the colour gradient located in the bottom right corner. Blue represents keywords that appeared earlier in time, and psoriasis is an example of an early‐appearing keyword. Yellow represents more recent keywords, and pyroptosis is an example of a recently emerged keyword that may become a new research direction.

### 3.9 Analysis of trends in keyword changes over time

For articles published between 2012 and 2022, the frequency of keyword occurrence was counted and ranked every 3, 2, 2, 2, and 2 years, respectively (Figure 6C). This ranking yielded a trend chart of keyword popularity over the past 11 years, where the curve fluctuation reflects changes in rankings. The keyword pyroptosis shows an upward trend. The empty circle symbolizes the timeframe in which the keyword initially emerged in research within the last 10 years, whereas the filled circle signifies the timeframe in which the research on this keyword concluded.

Regarding IL‐18 research, pyroptosis research is on the rise, with no decrease in popularity. Psoriasis research is also increasing. As of 2012–2020, psoriasis did not appear in the top 50 keywords. However, in 2021–2022, psoriasis entered the top 50 for the first time, indicating that IL‐18 research and psoriasis have gradually increased in recent years, and their popularity is on the rise.

### 3.10 Mapping gene co‐occurrence clusters: Insights from VOSviewer

The VOSviewer software was used to create a visualization map by conducting a co‐occurrence clustering analysis on genes associated with a specific study. Figure 6D includes genes that only occurred at least 70 times, and authors who met the criteria were also included. Every node comprises a circular shape and a label, where the circle size is directly proportional to the gene's occurrence frequency, and the thickness of the connecting lines among circles is directly proportional to the strength of the gene relationship. Clusters are formed by nodes of varying colours, with each colour representing a distinct gene clustering domain. The red cluster belongs to the domain of immune regulation and cell apoptosis, with IL‐1β being the most popular. The green cluster is in the domain of cytokines and immune regulation, with IFN‐γ being the most popular. The blue cluster represents the domain of the immune system and inflammation, with IL18 being the most popular (Figure 6D).

### 3.11 GO enrichment analysis

Figure 6E presents the results of the GO functional enrichment analysis. A bubble chart plots the top 10 *p*‐values of IL‐18's involvement in BP, molecular functions (MF), and cellular components (CC). In BP, proteins are mainly involved in the positive regulation of cytokine production; in CC, proteins are mainly related to the external side of the plasma membrane; in MF, proteins are mainly related to signalling receptor activator activity.

### 3.12 KEGG pathway enrichment analysis

Following the KEGG pathway enrichment analysis, 163 pathways were found to be significant. From these pathways, the top 10 pathways were chosen to create a bubble chart (Figure 6F). The findings suggest that IL‐18 research is primarily pertained to the pathway of interaction between cytokines and cytokine receptors.

### 3.13 Analysis of PPI networks

Using the Citexs big data platform, the top 100 proteins ranked by appearance frequency in the article were imported into the STRING platform (with a minimum occurrence of 164 times per protein) with ‘Homo sapiens’ as the species; high confidence (0.900) PPI network information was set. The network information was imported into the Cytoscape software, and the degree value was computed for each node.

After screening the genes and importing them into the STRING database, a PPI network was obtained, which contained 99 nodes, 489 edges, and an average node degree value of 9.88. The degree measure of every node was computed using the Cytoscape software, and a network diagram for core protein PPI was created by arranging the nodes in ascending order based on their degree values. The top 10 proteins are IL10, IL1B, IL6, RELA, IL18, STAT3, TNF, NF‐kappaB1, IL1A, and IL2 (Figure 6G); these proteins may be IL‐18‐related core proteins.

### 3.14 Mapping disease associations: Insights from co‐occurrence clustering analysis

The VOSviewer software was used to conduct a co‐occurrence clustering analysis, establishing a visual map by requiring a minimum occurrence of 85 for each disease. The circles and labels combine to create a node, where the circle size is directly proportional to the frequency of disease incidence, and the thickness of the circle connections is directly proportional to the intensity of the relationship among diseases. Clusters are formed by nodes of varying colours, with each colour indicating a cluster of diseases in various domains (Figure 6H). In the realm of inflammatory and autoimmune diseases, the red cluster stands out, with arthritis as the most prominent condition. The green cluster signifies diabetes and cardiovascular diseases, with diabetes mellitus being the most intense aspect. Finally, the blue cluster denotes neurological disorders, with stroke being the most significant disorder.

## 4 DISCUSSION

To the best of our knowledge, the present review represents the first bibliometric examination of the knowledge base and research boundaries of IL‐18. As of 31 December 2022, we identified 9753 articles in various journals authored by 54,493 individuals affiliated with 8223 institutions across 119 countries/regions. The annual publication volume serves as a pivotal metric for gauging the intensity and developmental trends of IL‐18 during specific periods. The growth trajectory of IL‐18 research commenced officially in 2014, and post‐2014, the publication volume of IL‐18‐related studies has increased substantially, indicating widespread attention to IL‐18.

Despite its discovery in 1995, IL‐18 has been acknowledged as a multifunctional cytokine inducing diverse biological effects associated with infection, inflammation, and autoimmune processes.^19^ The functional range of IL‐18 covers a range of conditions, including IBD. IL‐18 is involved in developing IBDs, such as ulcerative colitis and Crohn's disease.^20^ Its expression in intestinal tissues correlates with the activation of inflammatory responses and disease progression. IL‐18 is associated with chronic liver disease, participating in the inflammation and damage responses within liver tissues.^21^ Adult‐onset Still's disease—a systemic inflammatory disorder—is associated with IL‐18 in terms of its pathogenesis.^22^ The disease's pathophysiological processes might involve IL‐18. Eosinophilic granulomatosis with polyangiitis—typically associated with abnormal immune activation—may involve abnormal expression of IL‐18 in its pathogenesis. IL‐18 is implicated in the pathogenesis of rheumatoid arthritis, potentially influencing joint damage by regulating immune responses and inflammatory processes.^23^ Idiopathic thrombocytopenic purpura—usually associated with abnormal immune activity—may involve IL‐18 in its regulatory processes.^24^ The abnormal manifestation of IL‐18 is linked to the emergence and advancement of cancer, potentially affecting the biology of tumours by controlling immune responses and pathways related to cell growth.^25^ IL‐18 is associated with the development of cardiac conditions, such as heart attack and cardiac insufficiency. It may participate in the pathological processes of the heart by mediating inflammatory responses and abnormal activation of the cardiovascular system.^26^ Furthermore, recent studies have indicated that IL‐18 and its controlling factor, IL‐18BP, are linked to the emergence and progression of inflammatory skin conditions, such as psoriasis,^27^ atopic dermatitis,^28^ LE,^29^ rosacea,^30^ and pemphigus vulgaris.^31^

To investigate the progress and patterns of IL‐18 in the mentioned disease fields, examine current popular subjects, and anticipate future research paths, we conducted a bibliometric analysis of pertinent literature spanning from 2012 to 2022. The journals Nature and Nature Reviews Immunology have published the highest number of IL‐18 research papers and have the highest co‐citation frequencies (Table 4). Most IL‐18 studies align with the field of immunology and inflammation, as indicated by the dual‐map analysis (Figure 3). Journals specializing in immune system studies exhibit great interest in IL‐18 research because of its extensive regulatory function in both innate and adaptive immunity. Notably, the journals Nature, Nature Reviews Immunology, and Nature Methods collectively contribute to ~50% of the co‐citation volume in this field. This finding is attributed to the foundational research on IL‐18 published in these journals, garnering significant attention. The institutional spatial distribution map reveals that Shanghai Jiao Tong University has the highest publication output with 151 articles and 2730 total citations, making it the most prolific institution with high centrality and thereby playing a crucial bridging role in the institutional cooperation network. We examined the yearly and overall patterns in publications and citations for the top 10 most prolific nations worldwide. The findings indicate that China surpasses other nations in every aspect by a significant margin. Based on the institutional analysis, China is home to 90% of the top 10 most productive institutions (Table 2). Despite the United States having only 826 articles, its average citation rate (66.50) far exceeds that of China (19.78), closely followed by Australia (56.40). Although China is at the forefront of IL‐18 research, it is crucial to focus on improving the overall calibre of its published works.

Regarding the author distribution (Table 3), Kanneganti, Thirumala‐Devi, hailing from the United States, emerges as the most productive author, amassing a significant number of citations. In terms of published papers, Charles A. Dinarello, a native of the Netherlands, occupies the second position, whereas Mihai G. Netea, hailing from Germany, holds the third position in both publication and citation counts. The research team led by Kanneganti, Thirumala‐Devi, identified a novel member of the NLR (nod‐like receptor) family, NLRP12NLR, leading to in‐depth investigations. NLRs are a group of immune receptors found inside cells that can initiate the secretion of IL‐1b and IL‐18, as well as the cleavage and activation of caspase‐1.^32^ Thus, they play a role in controlling inflammatory reactions caused by infections and cellular harm. The team led by Dinarello, Charles A. elucidated the mechanistic role of IL‐18 and its binding protein in inflammatory diseases, providing updated insights into the biology of IL‐18 and its implications in human diseases.^7^ Similarly, the team led by Netea, Mihai G. conducted extensive research on IL‐18 in the context of inflammation and immunity. Their substantial contributions, particularly in highlighting the consequences of IL‐18 deficiency and leading to heightened phagocytosis, obesity, and insulin resistance, hold significant and far‐reaching implications for IL‐18 research.^33^

The occurrence and surge in popularity of keywords reflect the research hotspots in the field. High‐frequency keywords include ‘inflammation’, ‘pyroptosis’, ‘IL‐18’, ‘NLRP3 inflammasome’, ‘cytokines’, ‘oxidative stress’, ‘biomarkers’, ‘neuroinflammation’, ‘IL‐1β’, and ‘COVID‐19’. Through the intense citation bursts of keywords, we observed that literature from 2012 to 2014 has primarily focused on the fundamental structure and mechanisms of IL‐18, laying the foundation for a deeper understanding of its biological functions. Since 2015, there has been a significant rise in the occurrence of high‐frequency terms like ‘inflammation’, ‘cytokines’, ‘NLRP3 inflammasome’, ‘pyroptosis’, and ‘immune’. This finding suggests that the study of IL‐18's role in cellular immune responses and immune‐related diseases has become a prominent area of research.

Based on co‐occurrence analysis, IL‐18's mechanistic involvement in various diseases remains the most captivating subject of research. For instance, researchers are currently exploring and publishing papers on the role of IL‐18 in cancer immunotherapy and its association with the pathogenesis of cardiovascular diseases. The forefront of IL‐18 studies requires further confirmation through citation bursts of keywords, and emerging keywords related to ‘IL‐18’ may signify the continuous expansion and deepening of this field. Additionally, the persistent citation burst of the keyword ‘inflammation’ since 2012 suggests that IL‐18 remains a hotspot for future research in various inflammatory reactions. This finding indicates that, beyond disease‐specific studies, the regulatory role of IL‐18 in overall inflammatory diseases is also a highly scrutinized direction.

Despite certain limitations in current research, such as the potential exclusion of some IL‐18‐related articles in the WoSCC database, the retrieved literature from the WoSCC database exhibits the reliable characteristics of publication and citation. Additionally, the handling of specific data formats is more convenient in CiteSpace software, which is widely accepted in the field. Most bibliometric articles have utilized data from WoSCC.

It is important to note that, during the term extraction and cluster analysis process, some terms may exhibit high variability due to inherent limitations in the CiteSpace software. In the co‐occurrence analysis of keywords, it is advisable to use CiteSpace to integrate semantically related terms to more accurately reflect the knowledge structure and dynamic evolution of the research.

### 4.1 Limitations

This study has common deficiencies in bibliometrics. Although we want to fully include the papers in the research field as much as possible, due to the limitation of the software, we used the WoSCC database for retrieval, which may lead to incomplete included papers and certain bias. Second, we limit the language to English and the type of literature to articles and reviews, which may not be rigorous enough. Finally, we merged keywords according to the standardized steps of bibliometrics, such as the names of authors and institutions, but because the same name may have many expressions, the same author or institution may not be fully merged, which affected the accuracy of the research results.

## 5 CONCLUSION

In summary, there has been a consistent rise in the number of publications on IL‐18, indicating a growing interest and recognition of its importance in research endeavours. Collaboration networks among institutions and authors also highlight the importance of international cooperation in advancing IL‐18 research. Institutions such as Yale University and Shanghai Jiao Tong University have shown significant involvement in IL‐18 research, with the latter leading in publication count. Noteworthy authors such as Kanneganti, Thirumala‐Devi, and Dinarello have contributed substantially to the field. Analysis of journal publications reveals the interdisciplinary nature of IL‐18 research, with connections established across various fields. This interdisciplinary approach underscores the multifaceted implications of IL‐18 in different domains of study. Most IL‐18‐related studies are concentrated in the fields of biology and medicine, particularly in Immunology. Keyword analysis identifies emerging areas of interest such as pyroptosis, suggesting potential future research directions. Gene co‐occurrence clustering analysis highlights key genes associated with IL‐18 research, particularly those related to immune regulation and inflammation. PPI networks reveal core proteins like IL‐10, IL‐1β, and IL‐6, indicating their significance in IL‐18‐related pathways. GO and KEGG pathway analyses provide insights into the BP and pathways influenced by IL‐18, emphasizing its role in cytokine production regulation and interactions with cytokine receptors. IL‐18 research is closely linked to various diseases, particularly inflammatory and autoimmune conditions like arthritis and diabetes mellitus. Understanding these associations can lead to potential therapeutic interventions and diagnostic advancements.

This bibliometric analysis provides the visualization to the field of IL‐18, contributing to tracking the intellectual structure of specific knowledge domains and research fronts of IL‐18.

## 6 FUTURE DIRECTIONS

To further facilitate international collaboration among institutions and researchers, deepen the understanding of IL‐18 research, and expedite research progress. Given the emergence of novel keywords such as pyroptosis, continuous exploration of these new domains is warranted to unveil fresh insights into the mechanisms and therapeutic potential of IL‐18 in disease pathogenesis. In terms of clinical applications and therapeutic strategies, translating research findings into clinical applications and developing precision treatment strategies for IL‐18‐related diseases can offer new avenues for disease management and treatment. Additionally, further investigation into core proteins identified in PPI networks and pathways enriched by IL‐18 can elucidate their mechanisms of action and potential as therapeutic targets. Adopting interdisciplinary approaches to explore the multifaceted impacts of IL‐18, considering its implications in fields such as biology, medicine, and immunology. Exploring the potential of IL‐18 as a biomarker for disease diagnosis, prognosis, and treatment response prediction can pave the way for personalized medicine approaches.

## AUTHOR CONTRIBUTIONS

**Z.W.:** Conception and design; data analysis and interpretation; article writing; collection and assembly of data; collection of data and data analysis; Financial support; administrative support; article writing, and final approval of the article.

## FUNDING INFORMATION

This work was financially supported by the Scientific research projects of Shanghai HongKou Health Commission (2202–21) and the Scientific Research Start‐up Fund from Shanghai Fourth People's Hospital, School of Medicine, Tongji University.

## CONFLICT OF INTEREST STATEMENT

The authors declare that there is no conflict of interest.

## Data Availability

The data that support the findings of this study are available on request from the corresponding author. The data are not publicly available due to privacy or ethical restrictions.
